# Chlorate of Potassa

**Published:** 1872-12

**Authors:** L. P. Dodge

**Affiliations:** Farmington, Minnesota


### CHLORATE OF POTASSA.

ANNUAL ESSAY, BY L. P. DODGE, M. D., FARMINGTON, MINNESOTA.

[Read before the Minnesota State Medical Society.]

To the practicing physician there is no branch of the medical science of more importance than Materia Medica. His knowledge should be full and perfect, for in no field of science has there been more uncertainty or a more incongruous hetrogeneous mass of diverse and often contradictory opinions. A perfect understanding of this branch of the science is imperative, to become a successful practitioner.

If we understand perfectly the therapeutical action of the remedies we employ, both simple and in combination, the list would be simplified, and we should not be seen prescribing the same remedies in opposite conditions of the system. We should school our minds to systematically observe and record the peculiar action of the agents employed, and pay less attention to imaginative theories. From age to age the inquiring mind has traveled without a reliable chart or compass with which to guide its course. And imagination, ever active, has wandered uncontrolled from one hypothesis to another equally visionary, until the paths and bypaths have become so multiplied and intersect each other in so many directions, that Bichat has truly said, "Hence the vagueness and uncertainty our science presents at this day. An incoherent assemblage of incoherant opinions, it is, perhaps, of all the physiological sciences, that which best shows the caprice of the human mind. It is a shapeless assemblage of inaccurate ideas, of observations often puerile, of deceptive remedies, and of formulæ as fantastically conceived as they are tediously arranged."

There is no better time to discuss the action of different remedies, to compare notes, and to do away with the seeming inconsistencies of the therapeutical effect of remedial agents, than in meetings like this. Therefore, we think, that a certain portion of the time should be devoted to Materia Medica. Hence, I present at this time, for your consideration and discussion, "Chlorate of Potassa." The therapeutical effects of this agent are obtained by direct application and by absorption. When taken into the stomach, it imparts a cooling sensation to the mouth and throat; the circulation is somewhat depressed. Hence it has been classed by authors as a refrigerant, and from the increased action of the kidneys, diuretic. By some, it has been supposed, to excite hepatic action. Without doubt it does, but to what extent we are not prepared to state. When applied locally to ulcerative surfaces of the mucous membrane, as in ulcerative stomatitis, and many other diseases of the mucous membrane; and also to indolent ulcers of the integument, it has a stimulating action, shown by increased sensation of the parts and excited vascular action, which becomes alterative, and therefore salutary. Its most decided effects are obtained when taken into the system. Administered by the mouth, it is absorbed into the circulation, through which it comes in contact with those tissues for which it has an affinity in its action, viz: the mucous membrane, skin, and glandular system. That it is eliminated as chlorate of potassa by the kidneys, as proved by experiments of Wohler and Stehberger, is proof of its being absorbed. And "that it passes through the system as chlorate of potassa," is fatal to the hypothesis of the chemico-physiologists who fancied that it gave oxygen to the system, and was therefore well adapted for patients affected with scorbutic conditions, which were supposed to depend on a deficiency of this principle.

Chlorate of Potassa, we think, has a specific action on mucous membranes, the glandular and cutaneous systems. What that particular action is, or the *modus operandi*, we prefer to leave to more theoretical minds. We term its action alterative, because that term coincides with our ideas of its action, viz: that its salutary effects are produced by its being absorbed and coming in contact with the diseased tissue, as chlorate of potassa changing the morbid action into healthy.

Theories are very fine on paper, but the practical physician seeks for facts and these he frequently finds in direct opposition to the most ingeniously framed theory of the fanciful mind. In diseases of the mouth and throat, whether inflammatory or ulcerative, chlorate of potassa has a salutary effect. When administered at short intervals for twenty-four hours, the inflammation diminishes, as will be seen by the paler hue of the surface, less vascular turgesence, and diminished sensation. If ulcerative, the ulcers assume a healthy action and the fetor is removed. In diphtheria it is one of the most reliable remedies for the lesion of the throat. In no disease is its alterative action better shown. Give to an adult tablespoonful doses of the saturated solution every hour for twenty-four hours, and there will be a marked change in the general appearance of the diseased parts. The exudation will be diminished, the fetor removed, surface paler, the swelling diminished, the vascular action less, the sensation ameliorated, the skin becomes cool, the pulse less frequent; in fact a large per cent. of the incipient form of diphtheria requires no other remedy.

This may seem a very strong assertion, but from my experience in the treatment of this disease in a severe epidemic, which prevailed in 1864, in the central portion of New Hampshire, an epidemic which assumed all the forms, from a mild sore throat to the most malignant, sometimes overpowering the system in a few hours or laying the patient prostrate from loss of nerve power, I found no remedy that served me as well as did the chlorate of potassa, given either alone or, when required, in connection with other remedies. When it has failed I have not thought it a fault of the remedy, but that I did not administer it early, or was not persistent, or my dose was too small. In scarlatina anginosa, as well as in diphtheria, the administration of the chlorate will be found beneficial, given either alone or in connection with other remedies.

Not only in idiopathic diseases affecting the mouth and pharynx do we see its beneficial results, but also in syphilitic affections of those parts. We find in Braithwait's Retrospect, a report of a case of chronic syphilitic ulcers on the leg of long standing, which had resisted the influence of all the remedies usually employed, but yielded rapidly to the chlorate of potassa. A case of rupia syphilitica, which fell into my hands, after being treated by several professional men of more eminence than myself, yielded to the administration of chlorate of potassa with tonics, and the application of stimulating ointments.

From what has been said of its action on the throat, should we not expect similar results from its administration, upon the mucous membranes of other localities? In fact, we have proof of its remedial action in some forms of ulcerative action of the os uteri, and nasal passages, also when inhaled it frequently acts beneficially on the air passages.

The most pleasing results are seen when chlorate of potassa is given in ptyalism. And here we meet another of that class of medicinal agents, which administered, either in connection with or alternated, does not destroy the effects of the remedy given, but counteracts the morbific action. For example, when opium is administered or any of its preparations, its bad effects may be prevented by administering bromide of potassium at, or near, the same time. If a patient is suffering from nausea, vomiting, or vertigo, the result of opium, it is readily removed by the bromide of potassium, and the anodyne and soporific action of the opium are not impaired but rather increased.

The first action of mercury on the system is that of a stimulant to the mucous membranes and glandular structure, shown by an increased discharge of mucus and more abundant flow of the saliva and bile. This primary effect may be more salutary, but if carried too far it becomes morbific and then we find that the whole mucous membrane and glandular system is diseased, the secretions are abnormal, and we have the disease known as ptyalism.

Here we have just the condition for the administration of chlorate of potassa, in fact it is a remedy employed by a large portion of the profession.

On what grounds is it employed? Does it act chemically on the mercury? If chemically, there must be new compounds formed with the mercury and chlorate of potassa, each of the new compounds has the same base as before, viz: mercury and potassa. But all the preparations of mercury produce ptyalism. Why should not the new compound still aggravate the disease? If there is a chemical change, where does it take place, in the solids or fluids? For none will doubt but that both remedies are taken into the system, hence it must take place in one or the other, but it does not seem reasonable to suppose that the tissues are continually undergoing a chemical change regardless of any vital law.

If in the fluids, or in other words, the circulation—and here we must ignore all vital laws—convert the heart and blood-vessels into one grand retort, who can tell the changes and reactions that will take place after each dose of medicine? How shall we graduate our doses, so as to produce the desired chemical change? Again, does it follow that salts which are incompatible in the laboratory, should still continue to be so when taken into the system and have become subject to different influences and laws? But, from experiments made by Dr. O'Shaughnessy and others, chlorate of potassa passes undecomposed into the urine. Therefore there can be no chemical change, which we believe to be true; then chlorate of potassa must act directly as chlorate and its action to be alterative.

I think it has become a common practice with the profession, to give the chlorate of potassa to remove ptyalism, but I must go still further and say that it should always be given as a prophylactic, whenever it is necessary to give the mercurial for any length of time. Ptyalism is not the salutary effect of mercury any more than the discoloration of the skin from the long continued use of nitrate of silver, or the colic produced by the continued handling of lead, but morbific and uncalled for in the treatment of any disease; and it must be mortifying to any respectable practitioner to have a patient exhibit to him a swollen, ulcerated, fetid mouth, the result of his gross negligence, or abuse of one of the best remedies in the whole Materia Medica ; an abuse which has done more to establish that species of quackery known as homœopathy than all the rest combined. It has been my practice, for the last three years, to administer the chlorate of potassa in connection with a mercurial, whenever I desired to give the latter for any length of time. I do not administer the chlorate at the same time that I do the mercurial, but at longer intervals, and nicety of dose is immaterial, a small quantity is sufficient. I cannot see but that I obtain the therapeutical effects of the mercurial as readily as before I gave the chlorate. For instance, in chronic congestion and induration of the neck and os uteri, I have found it necessary to administer some one of the preparations of mercury for some weeks, and I have always given my patients chlorate of potassa once or twice a day while taking the mercurial, and never have I failed to obtain the effects of the mercurial, viz : removal of the disease or discharge from the bowels peculiar to the action of mercury. In secondary and tertiary syphilis I have employed the same agents with similar results.

But still more in active inflammation, when I have given repeated doses of calomel at short intervals with an occasional dose of the chlorate I have obtained the desired effect of the calomel, but never produced the slightest symptoms of ptyalism.

This is a subject worthy the investigation of the medical profession, and one of most practical utility. If new to any of you it must suggest to your minds the importance of its investigation. From what has been said we conclude, that chlorate of potassa has a specific action on the mucous membranes and glandular tissues of the throat, that that action is alterative in nature; and that it not only removes ptyalism, but that it is a sure prophylactic.

